# The impact of Fogarty International Center research training programs on public health policy and program development in Kenya and Uganda

**DOI:** 10.1186/1471-2458-13-770

**Published:** 2013-08-21

**Authors:** Sara Bennett, Ligia Paina, Freddie Ssengooba, Douglas Waswa, James M M’Imunya

**Affiliations:** 1 Johns Hopkins School of Public Health, Baltimore, MD, USA; 2 Makerere University School of Public Health, Kampala, Uganda; 3 Independent Consultant, Nairobi, Kenya; 4 UNITID, University of Nairobi, Nairobi, Kenya

**Keywords:** Research capacity, Policy influence, HIV/AIDS

## Abstract

**Background:**

The Fogarty International Center (FIC) has supported research capacity development for over twenty years. While the mission of FIC is supporting and facilitating global health research conducted by U.S. and international investigators, building partnerships between health research institutions in the U.S. and abroad, and training the next generation of scientists to address global health needs, research capacity may impact health policies and programs and therefore have positive impacts on public health. We conducted an exploratory analysis of how FIC research training investments affected public health policy and program development in Kenya and Uganda.

**Methods:**

We explored the long term impacts of all FIC supported research training programs using case studies, in Kenya and Uganda. Semi-structured in-depth interviews were conducted with 53 respondents and 29 focus group discussion participants across the two countries. Qualitative methods were supplemented by structured surveys of trainees and document review, including a review of evidence cited in policy documents.

**Results:**

In the primary focal areas of FIC grants, notably HIV/AIDS, there were numerous examples of work conducted by former FIC trainees that influenced national and global policies. Facilitators for this influence included the strong technical skills and scientific reputations of the trainees, and professional networks spanning research and policy communities. Barriers included the fact that trainees typically had not received training in research communication, relatively few policy makers had received scientific training, and institutional constraints that undermined alignment of research with policy needs.

**Conclusions:**

While FIC has not focused its programs on the goal of policy and program influence, its investments have affected global and national public health policies and practice. These influences have occurred primarily through strengthening research skills of scientists and developing strong in-country networks. Further success of FIC and similar initiatives could be stimulated by investing more in the training of policy-makers, seeking to better align research with policy needs through more grants that are awarded directly to developing country institutions, and grants that better incorporate policy maker perspectives in their design and governance. Addressing structural constraints, for example supporting the development of national research agendas that inform university research, would further support such efforts.

## Background

Many international and national agencies and foundations invest in the development of health research capacity in low and middle income countries [1,2]. For many such agencies the ultimate aim of this endeavor is to improve the health status of populations in these countries through scientific knowledge that creates new medical technologies and improves the design and delivery of health programs and policies [3,4]. However there is a long and perhaps tenuous causal chain between investment in individual researchers and the intended policy and programmatic impacts. For example trained individuals may not stay in the research field or may not purposefully seek to use their research findings to influence policy and practice. Accordingly, many evaluations of research capacity investment have focused on the proximal effects of the intervention: the number of people trained and retained, the number of peer reviewed publications produced [5]. Very few assessments have sought to understand the impact of research capacity development on policy and the use of evidence in policy and decision making. Those that have examined this, tend to have done so in a relatively narrow way, for example considering the impact of research training grants on capacity for knowledge translation [6].

Our focus is the research training investments made by the Fogarty International Center (FIC) of the United States, National Institutes of Health (NIH). FIC began to make serious investments in research capacity development in 1988 through its AIDS International Training and Research Program (AITRP). Since that time it has initiated many additional research training programs, and FIC now runs a total of 17 such programs covering areas such as infectious diseases, chronic non-communicable diseases, bioethics, as well as programs defined not by field of study but rather by the type of investigator targeted (e.g. early stage investigators under the Global Health Program for Fellows and Scholars) [7]. The focused investments in research capacity made by FIC, have been bolstered by substantial investments in health research in low and middle income countries, particularly in HIV, by the National Institutes of Health, through its many Institutes and Centers. For example between 1982 and 2009 the NIH invested US$42 billion in HIV research (at home and abroad) [8]. While data on how much of this was spent in low income countries such as Kenya and Uganda are not available, this backdrop of significant and, until recently, growing health research investments created a potentially fertile ground for newly trained investigators.

This paper seeks to assess whether FIC’s long term investments in scientific capacity in Kenya and Uganda had influenced national policy or practice in any way, and if so how. The findings reported here were part of a broader study examining the long-term effects of FIC support in Kenya and Uganda. At the outset of the study, based upon a review of the relevant literature we developed a conceptual framework that traced the potential channels through which investment in individual scientific training could accumulate over time to drive broader changes in organizational and network capacity for research and ultimately in policy and practice. It should be noted that historically there has not been a strong focus within Fogarty on influencing policy and practice; indeed the knowledge translation movement was nascent at the time that FIC started its programs [9]. Nonetheless, it was quickly apparent in both study countries that impacts at the policy and practice level had occurred.

There is a growing body of literature that seeks to understand and develop frameworks for assessing, the ways in which research influences policy and practice [10,11]. We drew upon such frameworks in this work, but also approaches to evaluating the impact of capacity building [12]. We suggest research training programs may impact policy and practice through three primary routes:-

• Better preparing individual researchers to engage with policy and practice

• Strengthening alignment between policy maker evidence needs and the nature of research conducted

• The development of professional networks that link research and policy/practice worlds.

The findings section of the paper first presents evidence regarding the impact of FIC training on policy and practice, then seeks to explain how these impacts occurred by analyzing the causal effects of investigator training, in particular through the channels identified above.

## Methods

Two exploratory case studies were conducted in Kenya and Uganda, focused on the University of Nairobi (UoN) and Makerere University, respectively. Case studies are an appropriate method to investigate complex phenomenon in a real life situation. These two countries were selected as being ‘extreme’ cases [13] in the sense that FIC has been supporting capacity development in both countries since 1988 when the first AITRP program began, and there have been large numbers of researchers trained by FIC in both countries (approximately 135 individuals have received long term training in Uganda, and 82 in Kenya). Thus while these cases are unlikely to be typical of the impact that FIC has had on policy and programs in low and middle income countries, they present a good opportunity to identify such impacts and analyze how they have occurred.

Data for this particular analysis were collected through a number of complementary strategies, namely:-

• Semi-structured in-depth interviews and focus group discussions were conducted with a range of selected key informants in both Kenya and Uganda (see Table 1). Semi-structured interview respondents were purposively selected so as to reflect a range of different types of exposure to FIC (including former trainees, university leadership and policy makers). For trainees we focused on those who had had the most significant exposure to FIC; such individuals had typically received long-term training, and sometimes multiple trainings. Trainees whom we interviewed were drawn from across the many years that FIC had been active in each country, but as many of the early trainees had risen to positions of institutional leadership, older trainees were particularly well represented in our sample. Focus group participants were randomly selected from the group of long term trainees who were not interviewed. Focus group discussions complemented the in-depth interviews by (i) enabling the research team to engage with a broader array of trainees and (ii) by providing a vehicle through which to administer the structured questionnaire (see below). For both focus groups and in-depth interviews the discussion guide (see Additional file 1) included questions about what impacts if any former FIC trainees thought their work had had upon policy and practice in their country, how well they thought their FIC training had prepared them to engage with policy and how they had gone about ensuring the impact of research findings on policy;

• Participants in focus group discussions were also asked to complete a structured questionnaire (see Additional file 2). The last set of questions asked respondents to identify specific policy and programmatic impacts of their work;

• References included in recent national and global policies were reviewed to identify papers written by former FIC trainees. In semi-structured interviews respondents identified topics on which FIC trainees had conducted substantive research (notably HIV/AIDS and malaria). We identified recent policy documents from both the global and national level related to these topics, and reviewed their list of references for the names of FIC trainees from the case study countries. By focusing on recent reports alone, we were not able to examine the contribution that FIC trainees have made over time, but this approach provided a current snapshot, that encapsulated contributions across all generations of FIC trainees.

**Table 1:** Samples for FGDs and Semi-structured interviews

**Form of interview/respondent**	**Uganda interviewees**	**Kenya interviewees**
Focus group discussions	6 (subjects = 19)	3 (subjects = 10)
Principal investigators	5 (US and Ugandan)	3 (US and Kenyan)
FIC Trainees	6 (4 university-based and 2 non-university-based)	20 (7 based at UoN and 13 outside UoN)
Institutional leaders	5	5
Policy makers	4	5
Total N	39	42

The interviews and focus group discussions were conducted in Uganda during April-May 2011 and in Kenya during September 2011, by a team composed of both international and national researchers. The team started out by establishing which FIC trainees remained in the country ^a^, and sampling was based upon this list. For individual interviews no one in either country refused to participate, although it was frequently difficult to secure appointments with policy maker respondents and some of those originally selected had to be replaced with others. In both countries FGDs were more problematic and not all invited participants turned up. This meant that there were fewer recent trainees among our respondents, than originally envisaged.

Written informed consent was obtained from all participants in the study. All interviews were recorded. In Kenya interviews were transcribed and analysed using Atlas.ti. In Uganda, detailed notes were taken on all interviews. Due to budgetary constraints not all interviews in Uganda were transcribed, but rather the researchers relied on detailed interview notes. Transcripts and interview notes were coded manually so as to maximize involvement of the Ugandan researcher. In Uganda where an interview had not been fully transcribed but covered relevant material, the researchers consulted audio files where appropriate. A framework analysis approach was used building upon the literature review and conceptual framework previously constructed. Within the “policy and practice” domain, codes tracked examples of policy impact, trainees’ perception of how they were prepared to engage with policy makers, policy makers’ perception of the research done by trainees and their institutions, as well as gaps, the nature of research participation in technical working groups and relatedly policy networks. A similar coding structure was applied to analyse both of the data sets.

The study adheres to the RATS guidelines on qualitative research. The research was approved by the Johns Hopkins School of Public Health IRB, and by ethics review committees at Makerere University and the University of Nairobi.

## Results

### Trainee contributions to policy development

#### Trainee perspectives

Many Fogarty trainees cited ways in which their research had impacted policy, both nationally and internationally. Research conducted by FIC trainees had contributed to an array of changes in policy and global guidelines that are elaborated in Table 2. The greatest impacts have been in the fields where FIC has made significant investments (notably HIV), but TB, STI and maternal health policies have also been affected. Interviewees acknowledged that this important research occurred not solely as a consequence of FIC research training grants, but most respondents perceived FIC training support to be critical to the research and hence ensuing policy and programmatic changes. The scale and duration of FIC support was also perceived to be important in terms of enabling the development of a critical mass of researchers in specific areas, as discussed in the quotation below.

*I think it has definitely created what I could call champions of change and I can think of very critical people who have really shaped the face of HIV in this country, ………So I think they really have changed not only the institutional capacity but even in terms of policy that is going to make an impact. I am currently involved in the Partners Prevention trial that is showing that if you use Anti-retroviral treatment for an HIV negative individual it protects them from acquiring HIV. Out of the four sites we have in Kenya, three of the PIs on the sites [names trainees} were trained at the University of Washington through the AITRP. So I think in terms of making an impact to the sciences and implementing it, people like [name of trainee] has been very instrumental in bringing key stakeholders together to talk about what are the issues we need to do about PrEP [pre-exposure prophlyaxis] and so forth. And so even they have changed not just the capacity but policy and even actual delivery of services.* Former Trainee, Kenya

**Table 2:** Examples of FIC trainee related research influencing policy and programs

**Policy issue**	**Relevant FIC trainee research**
Uganda	
Infant prophylaxis during intrapartum and breastfeeding periods	During the late 1990s and early 2000s a series of publications by a FIC trainee and colleagues [14-16] established the effectiveness of nevirapine in protecting infants from contracting HIV during the intrapartum period and breast feeding. One of these papers [15] was described as “an historic breakthrough” in a letter to the Lancet [17]. Recently the team has studied the effects of providing a single dose of nevirapine versus administering it for six months and this, along with evidence from elsewhere has also influenced global guidance.
Male circumcision to prevent HIV/AIDS	Two randomized control trials involving multiple FIC trainees that examined the effects of male circumcision on HIV transmission [18], have been widely referenced (the 2007 article has been cited nearly 900 times). This program of research has changed international and national policies.
Community-based TB programs	Uganda has been seeking to strengthen its TB programs through the use of community based directly observed therapy (DOTS). An early study in one district argued for the feasibility and effectiveness of such a program [19], and subsequently the Ministry of Health adopted community-based DOTS. Recent small scale studies also by FIC trainees explored the use of lay health workers in such contexts [20].
TB diagnosis	Studies by faculty at Case Western, one of the US partners supported by FIC, in which a Ugandan FIC trainee played a critical part, reviewed the usefulness of the 3^rd^ sputum in the diagnosis of smear-positive TB [21]. This study contributed to changes in international recommendations by the World Health Organization in 2007, so that only 2 rather than 3 sputum smears are taken for the diagnosis of TB.
Alcohol use during pregnancy	Research by a FIC trainee on the prevalence of alcohol use in pregnancy raised concerns about the number of women drinking alcohol immediately before and during pregnancy [22]. The study has led to a campaign by Mulago hospital to reduce alcohol use during pregnancy and the MOH is also seeking to raise awareness about this issue. A follow-up study examining the effects of enforcing alcohol labeling was conducted, and at the time of the research, discussions were underway about the policy implications of the findings.
Kenya	
Pediatric HIV	Research by FIC trainees and colleagues on infant feeding, breastfeeding and HIV transmission [23-31] contributed significantly to WHO guidelines on breastfeeding. A follow-up study coordinated by the WHO and known as the “Kesho Bora” study, was a multi-country study of antiretrovirals to prevent mother-to-child transmission and this again helped to change policy internationally with guidelines developed for HAART for the mother and extended prophylaxis for the baby. One of the same FIC trainees also served on the WHO committee developing guidelines in this area.
Treatment of herpes in discordant couples	Research by a FIC trainee and colleagues at the University of Washington (also supported by FIC), with support from the Bill and Melinda Gates Foundation examined the effectiveness of acyclovir in reducing genital ulcers, and thus slowing the transmission of HIV [32-34]. The study demonstrated that while acyclovir was effective in reducing genital ulcers it did not slow HIV transmission. While it is difficult to demonstrate the direct translation of this finding into policy (as it is a ‘negative’ finding) several respondents cited this study as being of considerable policy significance.
Syndromic management of STIs	Studies by a FIC trainee and colleagues at the University of Manitoba helped demonstrate that health workers could undertake syndromic management of STIs [35-38], although other work by FIC trainees and collaborators [39] demonstrated the poor quality of care often provided.

In addition to the concrete examples of policy change described in Table 2, respondents also discussed their participation in policy processes. This frequently took the form of participation in technical working groups and contributions by FIC trainees were often significant. For example, one senior former Fogarty trainee drafted the recent Joint Inter-Agency Task Team Technical Review Mission of Kenya’s Prevention of Mother to Child transmission (PMTCT) and pediatric HIV/AIDS care and treatment^b^.

*So I belong to the technical working groups for most of the groups which are involved in pediatric care HIV treatment and PMTCT. So usually we review the work that is ongoing, so we are the people who actually draft the guidelines, the national guidelines, ……we meet constantly throughout the year just review what is new, what we need to change and what needs to change in terms of policy work.* Former trainee, Kenya

Respondents in Uganda noted that where national policy impact has occurred, sometimes, it has been triggered by international organizations (particularly the World Health Organization (WHO)) adopting the findings of published research. For example, significant frustration was expressed about how long it took to get Ugandan research findings on male circumcision translated into policy.

*The typical example is about male circumcision results. It took now about three years for the Ministry to come up with a policy and now we are moving from policy to implementation. And this is an intervention which is so effective……But you can see there are other people, other countries, who never participated in the research who have already taken up this innovation and put it into practice.* Former trainee, Uganda

In Kenya, as in Uganda, there was a sense from respondents that in order to change domestic policy it was often necessary to seek changes in international policies and guidelines first.

*With PMTCT it was more of a focus on change in policy by [names two FIC trainees] … I’d say it really started at the WHO level. There, special task forces that were convened that involved investigators at that level, then with the WHO recommendations that eventually filtered down to the country level.* Former trainee, Kenya

*So my contribution may not be at national level - influencing policy documents here in the country but my contribution at the local level is more with the trainees that come through me and then with my colleagues here and at the international level* Former trainee, Kenya

#### Policy maker perspectives

Policy maker perspectives on the contribution of FIC trainees to policy development in-country varied according to the nature of the responsibilities of the policy maker. Policy makers who were responsible for communicable disease control, and particularly for HIV/AIDS and TB, typically perceived FIC trainees to have made a major contribution and described close ties between policy and research worlds. In contrast, those policy makers who had broader policy functions, for example who worked in Ministry of Health policy and planning divisions, appeared to have less engagement with the research community in general, and often were unaware of the FIC program.

*We work really very closely with the university now. They have the time, they have the knowledge and skills. Probably all we need to do is identify the research question or point out our operational difficulty*. Policy maker and former FIC trainee, Uganda (focused on communicable diseases)

*There are a few occasions when the research community prepares a presentation or a paper, and they come here, they request for audience, we give it to them and they disseminate their findings. But these are few instances……………..Often we don’t go to [the University] for issues of policy as we don’t have the money to pay them*. Policy maker, Uganda (focused on broader policy functions)

In Kenya, several policy makers referred to a tendency for universities and other academic institutions to conduct “academic” research which they contrasted with more operationally or practically oriented research. While policy makers in this country acknowledged the existing stock of capable epidemiological researchers, they articulated a need for other types of skills such as health economics.

*[…]we can see how well selected, well thought out operational research can affect policy as opposed to purely academic research. I can talk about mutation of DNA but it will take a long time for it to find relevance to change policy, you know? Yeah* Policy maker, Kenya

*[…]randomized clinical trials, I think we have done well there. The areas that I think we haven’t done very well in the health sector that is important for policy is health economics, we haven’t done much there, the issue of costing and cost effectiveness, we haven’t done very well.* Policy maker, Kenya

#### Citation analysis

We also explored the impacts FIC trainees had on health policy in their countries and globally through an analysis of citations in key policy documents (Table 3). The upper half of the table shows the number of citations of work by former FIC trainees that were included in key recent global norms and guidelines, relevant to FIC trainee research interests in the case study countries. The lower half of the table presents a similar analysis for recent national policy documents in Uganda and Kenya. In both countries many of the national level policy documents were not well referenced and largely referred to other government reports (as opposed to scientific papers with named authors). The analysis supports the respondent observations above, as it appears that FIC trainee research was more frequently referenced in global reports, particularly in recent global guidelines on HIV/AIDS, than it was in national policy documents.

**Table 3:** Number of references to publications of FIC trainees in specific global and national policy documents

**Uganda**						**Kenya**					
**Policy/guideline**	**Date**	**Total # Refs**	**Total # refs with FIC trainee authors**	**# Refs lead authored by FIC trainees**	**# FIC trainees as co-authors**	**Policy/guideline**	**Date**	**Total # Refs**	**Total # refs with FIC trainee authors**	**# Refs lead authored by FIC trainees**	**# FIC trainees as co-authors**
**Global policies/guidelines**											
Operational guidance for scaling up male circumcision services for HIV prevention (WHO/UNAIDS)	2008	18	1	0	5	Operational guidance for scaling up male circumcision services for HIV prevention (WHO/UNAIDS)	2008	18	2	0	5
Antiretroviral drugs for treating pregnant women and preventing HIV infection in Infants: recommendations for a public health approach (WHO)	2010	125	6	1	7	Antiretroviral drugs for treating pregnant women and preventing HIV infection in Infants: recommendations for a public health approach (WHO)	2010	125	1	0	2 ^d^
**National Policies/guidelines**						**National policies/guidelines**					
Second National Health Policy (MOH)	2010	9	0	0	0	Kenya Health Policy 2012–2030 Final draft ^e^	2012	10	0	0	0
Uganda Malaria Program Review Report 2001–2010 (MOH)	2011	35	1	0	1	Towards a malaria free Kenya: National malaria strategy 2009–2017 ^f^	2011	16	0	0	0
National HIV/AIDS Strategic Plan 2007/8-2011/12: Moving toward Universal coverage (Uganda AIDS Commission)	2007	29	5	2	9	National HIV/AIDS Strategic Plan 2007/8-2011/12: Moving toward Universal coverage (Uganda AIDS Commission)	2009	15	0	0	0

### Preparation for policy engagement

In both Kenya and Uganda the large majority of FIC trainees remained in the country and thus were in a position to engage with domestic policy issues. It is not an aim of FIC training programs to develop the skills of participants in policy engagement, however most respondents in Kenya felt that FIC training had increased their preparation to engage in the policy sphere through building trainees’ technical competence and their confidence in their own skills. Some Kenyan trainees however also recognized their own limitations in terms of being able to engage with the policy process and the fact that their FIC-supported training had not prepared them for this.

*So I think for me it has come out that I can at least be able to do research but then how would I move it to policy? So there are certain issues that probably need to be addressed or done differently to be able to make impact to the health outcomes. I am not really trained to be able to do those kinds of things, that kind of research. Then you find yourself in a place where you feel sort of frustrated.* Former Trainee Kenya

This sentiment was echoed by many of the trainees in Uganda and was uniform across all trainee cohorts in both countries. Very few trainees in Uganda felt that their Fogarty training had adequately prepared them to engage in policy debates or to participate in processes intended to draw scientific evidence into policy. While many respondents acknowledged that FIC training could have been stronger in terms of preparing trainees for engagement with policy issues, there was also a sense of uncertainty as to whether additional training for researchers was really the key obstacle to greater research uptake by policy makers. Instead several former trainees pointed to the lack of capacity within the Ministry of Health in Uganda to process research, and simultaneously to the lack of leadership provided by Ministry staff on research and suggested that more training for policy makers was needed.

### Alignment of research with policy and programmatic needs

In Kenya both researchers and policy makers recognized the disjuncture between the kind of research that was often being conducted and the needs of policy makers. This gap was seen in part to relate to the nature of training that FIC trainees had received and their limited preparation in terms of skills in health policy or health systems. But it was also understood to be a more structural or systemic problem that training alone was unlikely to solve, and that required a concerted effort to address.

*One of the issues that was being raised by the Ministry was that: ‘all that you researchers ever do is research, you don’t focus on the areas that we want you to do research on, and then we never even know what you are doing’. Really they are also feeling that gap and I think there is need for training for them as much as for us so that we all understand where we fit in.* Former trainee, Kenya

*[…].lots of work which has been done which could affect the practice and policy but they never come down to the policy makers because of the interlink between the ivory tower and the policy makers. Because the policy makers make policies, the ivory tower is supposed to generate data although at times it generates data that never goes to policy makers.* Institutional Leader, Kenya

Further, outside of specific spheres (particularly HIV/AIDS, TB and malaria) some policy makers perceived university based researchers to be unresponsive to the needs of policy and decision makers, and even researchers recognized the gulf in communication:

*This is the unfortunate bit. Research in Uganda is investigator driven. How do we engage policy makers to have them on board before the research is done, while we are setting the research agenda?* Former trainee, Uganda

Researchers, at times, also perceived policy-makers to be less attuned to making use of research for decision-making. However, some informants in Kenya saw the potential for a more symbiotic relationship with policy-makers, as many of them - particularly the younger ones - have an increasingly strong research orientation.

*I think they have got a new crop of people there who have been sensitized, who are knowledgeable because many of them, even if they are not trained through Fogarty we have got many people who are trained through a program and they really do understand the importance of research and they are constantly engaged, like whenever there is a question arising, so they send it out to us now.* Former trainee, Kenya

Increasing the research orientation of policy makers was also perceived to be important by Ugandan respondents. In Uganda, FIC trainees under one of the AITRP grants had originally been selected by a panel composed of representatives of the US University, Makerere University and the Ministry of Health and during this period a number of government officials received FIC training. However over time the Ministry’s role had dropped away and in the view of some this had undermined the policy and programmatic relevance of the FIC program and ultimately the research conducted.

*[…] so it goes back to this program should serve the MOH. I don’t know how this happened but the MOH is no longer involved.* Policy maker, Uganda

### Development of policy networks

In Kenya, for some respondents, there was a sense that FIC had enabled the development of their professional networks in ways that facilitated policy engagement. This was particularly true where policy makers or other government officials had received FIC training, but such networks also developed through training students who later went on to policy or programmatic roles.

*I think it’s very important to have people from the government side who are trained. X [former FIC trainee] has made a big difference…….I think part of the success of the HIV program, STD program compared to other ministry programs is that you have people like X who are trained in epidemiology and so you are evidence based, you are monitoring, the evaluation is not like sort of, this big crisis, you are able to look at it, okay! This is n, and we only reached 70% of n, what are the problems?* Institutional leader and former Trainee, Kenya

In Uganda many Fogarty trainees have now progressed into senior positions both within the University, but also outside it: for example, one is a member of parliament, others are at WHO, several work within the Ministry of Health, particularly in the infectious disease sections. This diffusion of trainees had created epistemic and policy networks [40] that facilitated impacts upon policy, as one trainee described it:

*Remember, Fogarty has been around since the 1980s when the [HIV/AIDS] epidemic started so now those people have grown up into more prominent positions, so they more visible, more senior, more likely to be consulted for their opinion…whereas some others [funders] have just recently joined*. Former trainee, Uganda

*If you know the people who are in the antiretroviral sub-committee for the Ministry of Health, most of them are Fogarty…not all but some have Fogarty experience. For us we are in the PMTCT track, we give advice and technical support.* Institutional Leader, Uganda

Particular individuals who had received Fogarty training and then returned to the Ministry of Health and ultimately reached relatively senior positions within the Ministry were frequently referred to by respondents in both Kenya and Uganda and appeared to have been key connectors, linking policy and research worlds. In Uganda respondents described the ties that linked them to these particular individuals both as the fact that they had shared or similar training experiences, but also that they continued to meet informally particularly through the Uganda Society of Health Scientists (a society established under one of the FIC grants). In Kenya, Technical Working Groups convened by the Ministry provided a means for researchers to connect to FIC trainees now working in policy positions. In particular FIC trainees are particularly active on the Technical Working Groups convened by the National AIDS and STI Control Program on PMTCT and Pediatric HIV.

## Discussion

There is fairly widespread agreement concerning the factors that support or inhibit the use of evidence in the policy process: personal contact between researchers and policy makers, timely and relevant research, clear research summaries, and good quality research all support the use of research evidence, whereas the absence of these factors undermines it [41]. This study has revealed a number of important ways in which the Fogarty investment in research training in Kenya and Uganda has affected health policy and practice in those countries. The primary manner in which Fogarty training has had an impact on policy and practice is through strengthening the technical skills of researchers, and hence the quality of the research produced and relatedly researchers' confidence in engaging in scientific debates. Former FIC trainees typically recognized that their Fogarty training experience had otherwise done little to prepare them directly for policy engagement, and indeed this was not an objective of the program.

In the scientific fields where FIC has focused much of its support, such as infectious diseases, and especially HIV/AIDS, our case studies uncovered strong networks and close personal contacts between FIC trainees and policy makers. Frequently these networks were strongest when they centered on policy-makers who had also benefitted from FIC training. Networks outside of the core FIC areas of focus appeared much weaker, and policy-makers identified a number of other skill sets (such as health economics) which were sorely needed. While this evidence suggests that FIC research training investments had substantial impact on the development of epistemic communities in countries, it is unlikely that the impacts would have been as great, if it were not for concurrent investments by the National Institutes of Health, the US government, and the broader global community in research on high priority diseases particularly HIV/AIDS, TB and malaria. The context of rapidly increasing funding for HIV/AIDS research, and scale up of services to tackle these and related diseases presented fertile ground for well-qualified researchers in these fields to engage with policy makers and practitioners. A further factor contributing to the impact FIC programs had on the development of epistemic communities was the long duration of support, which allowed inter-generational linkages to occur. For example, since FIC started providing research training support 25 years ago, senior trainees have grown into decision-making positions, and have supported policy engagement among more junior FIC trainees through acting as role models and facilitating personal connections.

Issues concerning the capacity of policy-makers to process evidence have been raised less frequently in the evidence-to-policy literature, with some notable exceptions [42], however this study suggests that training for policy makers may be a critical and somewhat neglected contribution. Not only can such training help enhance their appreciation of research evidence, but policy maker participation in training programs can also help bridge research and practice communities. Policy makers who had received research training informally played a role similar to that commonly attributed to knowledge brokers [43].

While it is clear that many FIC trainees were routinely engaged in policy making processes either through technical committees, or informal exchanges with policy makers, many researchers expressed frustration at their inability to get local research findings translated into policy change, without prior changes in global level policies and norms. Both, researchers and policy makers recognized systemic obstacles to better alignment of research with policy-maker needs for evidence. Interactions with policy-makers were seldom systematic or institutionalized, but more driven by individual relationships. Additionally, respondents described how research was “researcher-driven” rather than being driven by policy needs. Current funding structures, and in particular the limited amount of domestic support for health research, which leads to reliance on international donors for research funding, were understood to be an important part of this problem. Financial incentives mean that researchers are more likely to be responsive to evidence needs determined by international or foreign funders, than their own governments.

### Limitations

Much of the evidence on impact comes from the voices of the researchers themselves, who may be inclined to over-state the nature of the impact that their own research has had [44]. While we have triangulated our interpretation of policy impacts through interviewing policy makers as well, our sample of interviewees was biased towards researchers. The citation analysis provides more objective measures of research impact, while our qualitative data collection casts light on mechanisms for influence.

## Conclusions

The long-term focused investments that FIC has made in scientific capacity development have had considerable policy and programmatic impact, particularly around HIV/AIDS and other related topical areas, even though such impacts are not a stated objective of Fogarty programs. FIC trainees have typically not received training in knowledge translation or policy influence, but respondents did not view this as being the binding constraint upon more evidence-informed policy. Instead, the research points to a number of strategies that appear promising in terms of reinforcing the policy and programmatic impacts of capacity development initiatives. These include:-

• *Training government officials in health research and supporting their linkages to the research community* – training can help develop stronger research skills within government, but also coalesce networks that traverse policy and research fields. In the early days of one AITRP program in Uganda, the program committee for selecting trainees included Ministry of Health officials, and this meant that it was not uncommon for government officials to be sent for training. Ideally such an approach should be supplemented by other strategies that reinforce the knowledge brokering role of such individuals, such as support to local forums or networks that bring researchers and policy makers together.

• *Promote greater alignment of research with country needs* - the challenge of better aligning the types of research conducted with MOH needs is in some respects already being addressed by FIC. Increasingly FIC is encouraging research institutions in the South to be the principal investigators (and main recipients) of FIC grants. Enabling research agendas to be determined in-country should help support more policy relevant research. In addition mechanisms to better integrate policy makers into the advisory groups and governing structures of research capacity development grants may strengthen alignment of such programs with country needs.

The ESSENCE group ^c^, a group of research funders (including FIC) who are committed to strengthening research capacity in low and middle income countries, is also exploring mechanisms to encourage greater harmonization and alignment of research funding: this study speaks to the importance of this initiative.

Finally, while major investments have been made in developing scientific capacity, it is rare that such initiatives are properly evaluated, and the metrics to assess them typically focus on short-term easily measurable indicators (such as numbers trained), rather than more important but longer term effects [45]. There is currently growing interest in approaches to assess the societal impact of research [46], whether this is in terms of changes in policy or practice, or new technologies. Our case studies demonstrate that investment in public health research training can ultimately translate into changes in policy and practice, and in the future this should form part of a more systematic approach to evaluating scientific capacity initiatives.

## Endnotes

^a^ In Uganda 135 individuals had received long term training, of which we could trace the whereabouts of 126. 113 of these individuals were still in Uganda, and 66 of them worked for Makerere University. In Kenya, 82 individuals had received long term training, of which we could trace 72. Sixty of these individuals were still in Kenya with 41 of them employed by the University of Nairobi, the Kenya Medical Research Institute, or the Kenyatta National Hospital.

^b^http://nascop.or.ke/library/pmtct/JRM%20report%20final.pdf

^c^http://www.who.int/tdr/partnerships/initiatives/essence/en/

^d^ As members of the Kesho Bora study group

^e^ Available at: http://www.medical.go.ke/images/stories/downloads/kenya%20health%20policy.pdf

^f^ Available at: http://www.c-hubonline.org/sites/default/files/resources/main/Kenya_National_Malaria_Strategy_2009-2017.pdf

## Competing interests

The authors declare that they have no competing interests.

## Authors’ contributions

SB is the Principal Investigator in the overall study and proposed the scope for this article. LP and SB designed the study and were engaged in conceptualizing and preparing the first draft of this paper, incorporating other authors’ comments, and preparing for publication. FS, DW, and JM were engaged in editing and providing comments on the draft. All authors were involved in the data collection and analysis for the case studies. All authors read and approved the final manuscript.

## Pre-publication history

The pre-publication history for this paper can be accessed here:

http://www.biomedcentral.com/1471-2458/13/770/prepub

## Supplementary Material

Additional file 1 Case study interview guides. Click here for file

Additional file 2 Structured survey instrument. Click here for file
