# Supplementary material for: The impact of Fogarty International Center research training programs on public health policy and program development in Kenya and Uganda
Source: BMC Public Health. 2013 Aug 21;13:770. doi: 10.1186/1471-2458-13-770 (PMC3851767; doi:10.1186/1471-2458-13-770)
Supplement: Additional file 1 — Case study interview guides. [file 1471-2458-13-770-S1.docx]

# Data Collection Tools

1. In-depth Interview Guide for PIs^[[1]](#footnote-1)^
2. Please can you tell me a little about the history of this grant and how you first became involved in the grant program?
3. Can you describe the primary activities that have been implemented under this grant program in terms of trainings and other forms of support to capacity development?

*Probes*

*- What is the relative importance of short term versus long term training over the course of the grant?*

*- How have you determined what array of capacity development activities to implement?*

*- How are trainees selected?*

1. What role do the different institutions (US based institutions, LMIC institutions) play in terms of planning and implementing the grant?
2. How has support provided by Fogarty related to other sources of support for scientific capacity development, either through the NIH or through other funders such as the Wellcome Trust or Sida or IDRC?

*Probes*

- *Have scientists supported by Fogarty also been successful in securing other NIH grants?*
- *Are there other funders who have been actively supporting capacity development at the focal institution, and if so what kind of support have they provided?*

1. In your view what have been the main capacity development impacts of the grant in-country?

*Probes*

*- Has the grant contributed to a critical mass of scientists within the relevant field?*

*- Has the grant contributed to new teaching programs?*

*- Has the grant triggered any particular organizational development?*

1. Are there any particular success stories that you can tell me about concerning how FIC trainees have contributed to improvements in health policies, programs or clinical practice in the country?

- *Are there any particular trainees who stand out in your mind as having really benefitted from the program, and also having given a lot back to their own university, and whom you think it would be good for us to interview?*

1. Focus Group Guide for FIC Trainees

*Before we start, I would like to remind you that there are no right or wrong answers in*

*this discussion. We are interested in knowing what each of you think, so please feel free*

*to be frank and to share your point of view, regardless of whether you agree or disagree*

*with what you hear. It is very important that we hear all your opinions.*

*You probably prefer that your comments not be repeated to people outside of this group.*

*Please treat others in the group as you want to be treated by not telling anyone about*

*what you hear in this discussion today.*

*Let's start by going around the circle and having each person introduce himself or herself and tell us how long ago you did FIC training, what partner organization your training was associated with, what degree you earned through it, and how many years you have been at this University.*

*(Members of the research team should also introduce themselves and describe each of*

*their roles.)*

1. **FIC experience**

What were the strengths and weaknesses of your FIC experience?

***Possible Probes***

- *mentorship, organizational support, networking, structure of FIC training program*

1. **Organizational Capacity & Performance**

I would like to turn now to the question of how FIC support to training at this University has affected the overall capacity of this University. So we are not interested in how the training contributed to your individual skills or your research portfolio, but rather how FIC support may have impacted the University’s ability to secure research funding, develop its own teaching, or conduct scientific research etc?

***Possible Probes***

- *What effect, if any, did FIC have on your University’s ability to secure research funding?*
- *What effect, if any, has the FIC partnership had on the University’s teaching capacity, such as curricula development, recognition of leadership in teaching?*
- *Has FIC contributed to the development of a critical mass of scientists at your University?*

1. **Relative contribution of FIC versus other players**

Presumably there have been several other funders and partners that have contributed to capacity development at this University. How significant a player do you think FIC has been in terms of supporting the development of scientific capacity here compared to other actors?

***Possible probes***

- *Who are the other actors?*
- *What have they contributed?*
- *Has the long time frame over which FIC has contributed made a difference?*
- *Has there been anything particularly unique or valuable about FIC’s contribution?*

1. **Intervening Factors/Obstacles**

In your view what are the main constraints to further strengthening health research capacity at this organization, and do you think these are issues that Fogarty or similar programs can or should address?

1. **Network Capacity & Performance**

To what extent do you think that there is a professional network that has emerged around those involved with the Fogarty training program, for example do you find that you have regular interactions with other FIC trainees, or researchers at the US parent organization?

***Possible probes***

- *How strong are these networks vis a vis the other professional networks that you are a part of?*

1. **Research to policy**

What impacts, do you think your collective research has had upon health policy development and health care practice in this country, and more broadly upon the health of the population?

***Possible probes***

- *Are there any specific examples you can cite of how your research has made a difference?*
- *What opportunities have you had to ensure your research results reach policy and program audiences?*
- *How has FIC training prepared you to engage in dialogue with research users; to disseminate your research findings; to become engaged in the policy process?*

1. In-Depth Interview Guide for FIC Trainees currently employed in University Setting
2. Please begin by telling me a bit about how you became connected to the FIC training program, how you ended up in your current position and the extent to which you continue to conduct research in your current position?

**Individual Performance & Behavior:**

1. What were the strengths and weaknesses of your FIC experience and how did your FIC experience influence your career development?

***Possible probes***

- Mentorship
- Organizational support
- Career advancement/promotion
- Opportunity for collaboration researchers in-country, regionally, and internationally
- Ability to publish in peer-reviewed journals
- Ability to attract funding

1. What are the factors which motivate you to continue your career and to remain at your current organization?

***Possible probes***

- If the respondent has moved from one organization to another – then what prompted that move, what are the issues that have guided their career development pathway?
- Other career options considered
- Relationship between motivation to stay in current career and FIC training

**Organizational Capacity & Performance**

1. I would like to turn now to the question of how FIC support to training at this University has affected the overall capacity of this University. So I am not interested in how the training contributed to your individual skills or your research portfolio, but rather how FIC support may have impacted the University’s ability to secure research funding, develop its own teaching, or conduct scientific research etc?

Possible probes

What has been the impact of FIC support on:-

- Ability to secure research funding
- Teaching capacity
- Curricula development
- Recognition of research leaders
- Critical mass of research scientists (what other factors could have influenced the development of a critical mass?)
- Improved career development options

1. In your view what are the main constraints to further strengthening health research capacity at this organization, and do you think these are issues that Fogarty or similar programs can or should address?

***Possible probes***

- Lack of a strong research culture
- Weak systems of management and finance
- Lack of research infrastructure, labs etc.

**Network Capacity & Performance**

1. What effect has your participation in the FIC training program had on your professional network?

***Possible probes***

- Any interactions with other FIC trainees
- Researchers or professionals from other organizations, including at US parent organization, other international organizations.
- Relationships with mentors
- Professional organizations

**Research to policy**

1. What impacts, do you think research you have been involved in has had upon health policy development and health care practice in this country, and more broadly upon the health of the population?

- Are there any specific examples you can cite of how your research has made a difference?
- What opportunities have you had to ensure your research results reach policy and program audiences?
- How has FIC training prepared you to engage in dialogue with research users; to disseminate your research findings; to become engaged in the policy process

1. In-Depth Interview Guide for FIC Trainees Outside of University Setting
2. Please begin by telling me about how you became connected to the FIC training program, how you ended up in your current position and the extent to which you continue to conduct research in your current position?

**Individual Performance & Behavior:**

1. What were the strengths and weaknesses of your FIC experience and how did your FIC experience influence your career development?

***Possible probes***

- Mentorship
- Organizational support
- Career advancement/promotion
- Opportunity for collaboration researchers in-country, regionally, and internationally
- Ability to publish in peer-reviewed journals
- Ability to attract funding

**Network Capacity & Performance**

1. What effect has your participation in the FIC training program had on your professional network?

***Possible probes***

- Any interactions with other FIC trainees
- Researchers or professionals from other organizations, including at US parent organization, other international organizations.
- Relationships with mentors
- Professional organizations

**Research to policy**

1. What impacts, do you think your FIC training – and that of others in this country - has had, if any, upon health policy development and health care practice in this country, and more broadly upon the health of the population?

***Possible probes***

- Are there any specific examples you can cite of how your technical skills or those of other FIC trainees have made a difference?
- How has FIC training prepared you to engage in dialogue with policy makers and practitioners and to become engaged in the policy process

1. In-Depth Interview Draft Guide for University Leadership
   - - 1. **General**: Please begin by telling me about your involvement, if any, with the FIC partnership and how it has changed over time?
       2. **Individual Performance and Behavior**: From your perspective, what are the effects of the FIC experience on your staff?

***Possible probes***

- Ability to attract funding
- Scope for career development
- Development of professional networks
- Motivation to stay in research
- The development of researchers in-country, regionally, and internationally
- Researchers’ ability to publish in peer-reviewed journals

1. **Organizational capacity and performance**:  From your perspective, what are the effects of the long-standing partnership supported by FIC on your University?

***Possible probes***

- For example, the University’s research portfolio and budget.
- The number of researchers; there being a “critical mass” of researchers
- The researchers and university’s ability to attract funding
- The number of research leaders
- The number and quality of research partnerships
- How has the FIC partnership influenced meeting the research goals stated by your University in its mission (probably also found online)?

1. **Organizational support**: Please describe the opportunities and challenges in conducting research at this university and how the FIC programs may have modified them.

***Possible probes***

- Lack of a strong research culture
- Weak systems of management and finance
- Lack of research infrastructure, labs etc.

1. **Relative contribution of FIC versus other players**

Presumably there have been other funders and partners that have contributed to capacity development at this University. How significant a player do you think FIC has been in terms of supporting the development of scientific capacity here compared to other actors?

***Possible probes***

- *Who are the other actors?*
- *What have they contributed?*
- *Has the long time frame over which FIC has contributed made a difference?*
- *Has there been anything particularly unique or valuable about FIC’s contribution?*

1. **Network capacity and performance**: What contribution, if any, would you say that FIC had to the development of professional networks, both among staff at the University but also with others outside the University?

- Please describe your interactions with the US parent university and other FIC partner universities, if any.
- Please describe whether and how your opportunities to collaborate with researchers from other organizations was influenced by the FIC partnership

1. **Research to policy:** What impacts, do you think health research conducted by University staff has had upon health policy development and health care practice in this country, and more broadly upon the health of the population?

***Possible probes***

- Are there any specific examples you can cite of how research from the University has made a difference?
- What links does the University have with policy-makers and advocates and how have they changed throughout the course of your FIC partnership?
- Are there any particular policy-makers who have had close connections with your department/institute and whom you think it would be important for us to interview?
- Please describe how the type of research conducted and its relevance to policy changed due to your University’s FIC partnership.

1. In-Depth Interview  Guide for Policy Makers and other Research Users
2. **Information needs**:

Please can you start by telling me a little about the nature of the job that you perform so that we have a better understanding of what types of needs for information you have.

1. **Information gathering preferences**:

To what sources (organizations and individuals) would you go for (i) technical advice and (ii) in-country research on HIV/AIDS/malaria/TB *[ask as appropriate to the respondent].*

***Possible probe***

- To what extent do you use University of Nairobi as a source of (i) technical advice and (ii) research? Why is this the case?

1. **Views on Health Research Capacity at University of Nairobi**

Please tell me about your thoughts on the capacity of University of Nairobi and its research team (and its capacity to conduct policy-relevant research) .

1. **Awareness of FIC training programs**

Are you aware of the support to training in health research that the Fogarty International Center has provided to University of Nairobi? If so, what are your impressions of what the training provided by this program has been able to achieve?

1. **Research to policy**

5.1 What impacts, do you think research conducted by University of Nairobi has had upon health policy development and health care practice in this country, and more broadly upon the health of the population?

***Possible probes***

- Are there any specific examples you can cite of how their research has made a difference?
- We are aware of recent changes in program/policy on issue X; in what ways, if at all, was University of Nairobi involved in the development of this program/policy?
- Additional probes might mirror the suggestions provided by trainees.

1. [↑](#footnote-ref-1)
