# Supplementary material for: The impact of Fogarty International Center research training programs on public health policy and program development in Kenya and Uganda
Source: BMC Public Health. 2013 Aug 21;13:770. doi: 10.1186/1471-2458-13-770 (PMC3851767; doi:10.1186/1471-2458-13-770)
Supplement: Additional file 2 — Structured survey instrument. [file 1471-2458-13-770-S2.docx]

# Case Study Questionnaire for FIC Trainees

The Fogarty International Center (FIC) at the National Institutes for Health (NIH) is dedicated to supporting and facilitating global health research conducted by U.S. and international investigators, building partnerships between health research organizations in the U.S. and abroad, and training the next generation of scientists to address global health needs. FIC has made substantial investment in Kenya over the years, and is interested in developing a more comprehensive understanding of the long term impacts of FIC research training programs including not only the outcomes in terms of the professional development and career paths of individual trainees, but the broader effects upon organizational capacity, knowledge production, and policy development among other things.

As a former Fogarty we would like you to complete this short survey concerning your career after your Fogarty trainee.

1. **Personal Information**

| Name |  |
| --- | --- |
| 1.1 Age | □ 20-29 □ 30-39 □ 40-49 □ 50-59 □ 60 or older |
| 1.2 Gender | □ Male □ Female |
| 1.3 Current Employer |  |
| 1.4 Current Position |  |
| 1.5 Years at organization | □ 0 to 5 years □ 6 to 10 years □ Over 10 years |
| 1.6 Please indicate what percentage of your time you approximately spend within the following categories in a typical year: | Research ________________________________ %  Teaching/mentorship ______________________ %  Policy and practice advice ___________________%  Clinical practice ­___________________________ %  Research management _____________________ %  University administrative duties ______________ %  Other (please explain) ______________________ %  TOTAL________________________________ 100 % |
| - 1. Please indicate which teaching courses you currently contribute to. | Course 1:___________________________________  Course 2:___________________________________  Course 3:___________________________________  Course 4:___________________________________  Course 5:___________________________________ |
| 1.8 FIC Training Period | From : ______________(Month, Year) To : _____________(Month, Year) |
| 1.9 Degree received upon completion of FIC training | □ Master’s degree (Specify field: ____________________)  □ Ph. D. (Specify field: ____________________)  □ Medical degree (e.g. MD)  □ Dental degree (e.g. DDS)  □ Other: Specify ____________________ |
| 1.10 Other advanced degrees | □ Master’s degree (Specify field: ____________________)  □ Ph. D. (Specify field: ____________________)  □ Medical degree (e.g. MD)  □ Dental degree (e.g. DDS)  □ Other: Specify ____________________ |
| 1.11 Please indicate source(s) of support for other advanced degrees, NOT funded by FIC | Source(s): _____________________________________ |

1. **Satisfaction with organizational environment**

| **Please indicate the degree to which you are satisfied or dissatisfied with each of the following aspects of your work** | **Very Satisfied** | | **Satisfied** | **Neither satisfied nor dissatisfied** | **Dissatisfied** | **Very dissatisfied** |
| --- | --- | --- | --- | --- | --- | --- |
| **2.1 Career Development Pathways/Professional Development** | | | | | | |
| My job security within this organization |  | |  |  |  |  |
| My prospects for promotion |  | |  |  |  |  |
| That my academic achievements will be duly rewarded in terms of promotion |  | |  |  |  |  |
|  | | | | | | |
| **Please indicate the degree to which you agree or disagree with the following statements.** | | **Strongly Agree** | **Agree** | **Neither agree nor disagree** | **Disagree** | **Strongly Disagree** |
| **2.2 Adequacy of remuneration** | | | | | | |
| High achievement on the job is reflected in our pay | |  |  |  |  |  |
| My job offers adequate pay compared with similar jobs | |  |  |  |  |  |
| The income I receive is a fair reflection of my skills, knowledge and training | |  |  |  |  |  |
| The income I receive more than covers my basic needs such as food, transport and accommodation | |  |  |  |  |  |
| With this job I have no worries about how to support myself and my family | |  |  |  |  |  |
|  | | **Strongly Agree** | **Agree** | **Neither agree nor disagree** | **Disagree** | **Strongly Disagree** |
| **2.3 Availability of resources & Bureaucratic efficacy** | | | | | | |
| This organization provides everything I need to do my job effectively | |  |  |  |  |  |
| My work is rarely disrupted due to bureaucratic processes | |  |  |  |  |  |
| There are adequate resources (office space, computer systems, libraries and reference resources) for me to work effectively | |  |  |  |  |  |
| I am often prevented from getting my work done effectively and efficiently by bureaucracy | |  |  |  |  |  |
| I receive sufficient assistance with grant management and administration | |  |  |  |  |  |

1. **Research experience (Please complete ONLY if you currently spend most of your time in a university setting)**

| **Research experience** | |
| --- | --- |
| 3.1Out of your whole career, how many years have you been in a position which involved conducting at least some research? | **Years** |
| 3.2 Approximately how many peer reviewed journal articles have you published? |  |
| 3.3 On how many of these peer reviewed journal articles were you first author? |  |
| 3.4 Approximately how many non-peer reviewed journal articles, program reports, and policy briefs have you published? |  |
| 3.5 Approximately how many times have you presented research findings at conferences? |  |
| 3.6 Since completing your academic training how many research grants have you been the principal investigator on? |  |
| 3.7 Since completing your academic training how many grants have you been a member of the research team on? |  |

1. **Research experience and satisfaction (Please complete ONLY if you currently spend most of your time in a university setting)**

| **Please indicate the degree to which you agree or disagree with the following statements** | **Strongly Agree** | **Agree** | **Neither agree nor disagree** | **Disagree** | **Strongly Disagree** |
| --- | --- | --- | --- | --- | --- |
| **Research Satisfaction** |  |  |  |  |  |
| 4.1 I make a significant contribution to the University’s research portfolio |  |  |  |  |  |
| 4.2 I make a significant contribution to the development of health policy within my country |  |  |  |  |  |
| 4.3 I make a significant contribution to the development of clinical practice within my country |  |  |  |  |  |
| 4.4 I am satisfied with the time I have available to conduct research |  |  |  |  |  |
| 4.5 I have autonomy to choose my research direction |  |  |  |  |  |
| 4.6 My organization’s leadership helps protect my time to conduct research |  |  |  |  |  |
| 4.7 My current research work is attractive compared to other available job opportunities |  |  |  |  |  |
| **Overall satisfaction** | **Yes, definitely** | **Probably** | **Not sure** | **Probably not** | **No, definitely not** |
| 4.8 If you had to decide all over again, do you think you would choose a research career at this organization? |  |  |  |  |  |
| 4.9 Would you recommend a research career at this organization to a colleague? |  |  |  |  |  |
| 4.10 Do you think you will look for a job outside your organization in the next year? |  |  |  |  |  |

5. Please describe any particularly significant impacts that your research and/or programs have had in the last 5-10 years.

a.

b.

c.

THANK YOU VERY MUCH FOR YOUR PARTICIPATION
